# Odor–color associations differ with verbal descriptors for odors: A comparison of three linguistically diverse groups

**DOI:** 10.3758/s13423-016-1179-2

**Published:** 2016-10-25

**Authors:** Josje M. de Valk, Ewelina Wnuk, John L. A. Huisman, Asifa Majid

**Affiliations:** 1 Centre for Language Studies, Radboud University, P.O. Box 9103, 6500 HD Nijmegen, The Netherlands; 2 Max Planck Institute for Psycholinguistics, Nijmegen, The Netherlands; 3 Donders Institute for Brain, Cognition, and Behavior, Radboud University, Nijmegen, The Netherlands

**Keywords:** Cross-modal associations, Olfaction, Color, Cross-cultural, Cross-linguistic

## Abstract

People appear to have systematic associations between odors and colors. Previous research has emphasized the perceptual nature of these associations, but little attention has been paid to what role language might play. It is possible odor–color associations arise through a process of labeling; that is, participants select a descriptor for an odor and then choose a color accordingly (e.g., banana odor → “banana” label → yellow). If correct, this would predict odor–color associations would differ as odor descriptions differ. We compared speakers of Dutch (who overwhelmingly describe odors by referring to the source; e.g., *smells like banana*) with speakers of Maniq and Thai (who also describe odors with dedicated, abstract smell vocabulary; e.g., *musty*), and tested whether the type of descriptor mattered for odor–color associations. Participants were asked to select a color that they associated with an odor on two separate occasions (to test for consistency), and finally to label the odors. We found the hunter-gatherer Maniq showed few, if any, consistent or accurate odor–color associations. More importantly, we found the types of descriptors used to name the smells were related to the odor–color associations. When people used abstract smell terms to describe odors, they were less likely to choose a color match, but when they described an odor with a source-based term, their color choices more accurately reflected the odor source, particularly when the odor source was named correctly (e.g., banana odor → yellow). This suggests language is an important factor in odor–color cross-modal associations.

**Electronic supplementary material:**

The online version of this article (doi:10.3758/s13423-016-1179-2) contains supplementary material, which is available to authorized users.

There appears to be a tight link between odors and colors. Odor identification is easier when an appropriate color is presented together with the odor (e.g., Blackwell, 1995; Davis, 1981; Zellner, Bartoli, & Eckard, 1991), as is odor discrimination (Stevenson & Oaten, 2008). For example, when a scented solution is appropriately colored (e.g., strawberry odor in red water), rather than inappropriately colored (e.g., strawberry odor in green water), it is easier to distinguish it from other odors (Stevenson & Oaten, 2008). Similarly, normally colored bacon or cheese is perceived to have a more intense and better-quality smell than bacon or cheese that is colorless or is inappropriately colored blue (Christensen, 1983). Even wine experts are heavily influenced by color in their judgments of wines (e.g., Morrot, Brochet, & Dubourdieu, 2001; Williams, Langron, & Noble, 1984).

Some studies have explored odor–color associations by asking participants to associate specific colors with odors. These studies seem to find consistent mappings grounded in general knowledge—such as between the odor of a banana and the color yellow (e.g., Demattè, Sanabria, & Spence, 2006; Gilbert, Martin, & Kemp, 1996; Stevenson, Rich, & Russell, 2012), although other associations appear less intuitive—such as between vinegar and pink (Stevenson et al., 2012) or between mushroom and blue (Spector & Maurer, 2012).

A recent cross-cultural study by Levitan et al. (2014) showed that people within a culture consistently link colors to odors, but across cultures there are substantial differences, suggesting at least some odor–color associations are learned. If so, this learning could have a statistical origin—that is, odors are directly linked to colors because odor–color pairs are repeatedly experienced together (e.g., banana odor → yellow color)—or they could have a semantic origin—for instance, odor–color pairs are associated via language (e.g., banana odor → “banana” label → yellow; cf. Spence, 2011).

The possibility that semantics might drive odor–color associations has not received much attention, and when it has, it has been discounted in favor of perceptual alignments (e.g., Deroy et al., 2013; Spector & Maurer, 2012)—that is, structural associations (Spence, 2011). People (in the West) find it difficult to name odors (e.g., Cain, 1979; Engen, 1982; Majid, 2015; Majid & Burenhult, 2014; Olofsson & Gottfried, 2015; Yeshurun & Sobel, 2010), which makes it seem unlikely that language could play a mediating role in odor–color associations (Gilbert et al., 1996; Levitan et al., 2014). If we cannot name odors, how could language be the basis of our mappings? Besides, odor–color associations exist even for complex odors that do not have a specific label (Gilbert et al., 1996; Levitan et al., 2014; Schifferstein & Tanudjaja, 2004), which could be interpreted as evidence against the semantic account.

However, even if a complex odor cannot be labeled veridically, this does not prevent the use of language: People might label the predominant component of the odor and subsequently base their color choice on that label (cf. Zellner, 2013). Several findings support this account. For example, colors assigned to unisex fragrances depend on whether the person thinks the fragrance is male or female (Zellner, McGarry, Mattern-McClory, & Abreu, 2007). In addition, when people correctly name a lemon odor as *lemon*, the odor is linked to the color yellow; but when the same odor is misidentified as *lime*, people associate the same odor with green instead (Stevenson et al., 2012).

## Linguistic strategies to describe odors

The way we talk about smells varies across cultures. Speakers of Western languages, such as English, almost exclusively describe odors by referring to their source—for instance, *smells like banana* (Majid & Burenhult, 2014). This contrasts with how we talk about color, texture, or sound—for example, where “basic” or abstract terms are used instead (e.g., *red*, *blue*, or *green* for color; *soft* and *smooth* for touch; *quiet* and *loud* for audition; etc.) (Levinson & Majid, 2014; Majid, 2015). But speakers of other languages, such as Jahai and Maniq, hunter-gatherer groups in Southeast Asia, do have abstract terminology for odors (Majid & Burenhult, 2014; Wnuk & Majid, 2014). These terms are not source-based, nor are they restricted to a narrow class of objects. The terms refer only to odors. In addition, they are monolexemic and psychologically salient, and therefore are classified as “basic” vocabulary (Burenhult & Majid, 2011; Levinson & Majid, 2014). In Jahai, for example, *ltpɨt* describes fragrant smells coming from various flowers, perfumes, and bearcats, whereas *plʔeŋ* describes smells coming from, amongst other things, blood, raw fish, and raw meat.

When Jahai speakers are asked to name odors in controlled experimental settings, they rarely use source-based descriptions; they rely on these abstract smell terms instead. In fact, they describe smells in a comparable manner to colors, unlike English speakers (Majid & Burenhult, 2014). This raises the question of whether the speakers of a language with abstract smell terminology would behave differently in an odor–color cross-modal mapping task. If odor–color mappings are based on internal generation of a label, then the speakers of a language using abstract terminology should not associate odors to colors in a systematic manner, since abstract terms are usually linked to multiple sources of different colors (e.g., banana odor → abstract odor term *ltpɨt* → ? ). If, however, odor–color associations are based on structural or statistical associations alone, we should see comparable odor–color associations across cultures (e.g., banana odor → yellow color).

To test these hypotheses, we compared Dutch, Maniq, and Thai speakers. Dutch, like English, does not have an elaborate smell lexicon, so speakers overwhelmingly rely on source-based descriptions to describe odors. Maniq, like Jahai, has a rich odor lexicon (Wnuk & Majid, 2014), so we hypothesized that speakers would use abstract smell terms to name odors and therefore would not conceptualize odors as belonging to concrete sources. As a result, we predicted Maniq speakers would be less likely than Dutch speakers to show reliable odor–color associations.

Dutch and Maniq speakers differ from one another in many respects. They speak different languages, but they also differ in the environments they live in (city vs. tropical rainforest), settlement patterns, economic organization (settled postindustrial vs. nomadic hunter-gatherer), schooling (literate vs. nonliterate), and so forth. Thus, any differences in cross-modal associations between these two groups could, in principle, be difficult to interpret. We therefore also included a third group of Thai speakers in our study. Like the Maniq, the Thai appear to have a more elaborate smell lexicon than Dutch speakers (see the Results section); but unlike the Maniq, the Thai tested here also live in an urban environment, with a modern postindustrial society, widespread literacy, and so forth. The Thai group, then, presents an additional test case for the hypothesis that abstract smell terminology should lead to less consistent and precise color choices to odors than source-based odor descriptors.

## Method

### Participants

We recruited 59 participants: 11 Maniq (five female, six male; *M*_age_ = 41.4 years, range = 20–65), 24 Thai (19 female, five male; *M*_age_ = 21.2 years, range = 19–23), and 24 Dutch (18 female, six male; *M*_age_ = 25.6 years, range = 20–60). The Maniq sample was smaller due to practical restrictions: There are only about 300 Maniq speakers, living in small nomadic groups scattered across a large area, which makes it hard to recruit participants. All participants gave informed consent and were paid for participation (and, in the case of the Maniq, given food provisions) according to local rates.

### Stimuli

The odor stimuli were ingestible real objects placed in an opaque squeezy bottle. The odor objects were commonly found in the Netherlands, in Thailand, or in both (Table 1).

**Table 1:** List of odor stimuli used in the experiment

Common Objects, Thailand	Common Objects, The Netherlands	Common Objects, Thailand/The Netherlands
Fermented Petai beans	Mustard	Banana
Dried durian	Licorice	Tobacco
Shrimp paste	Red wine	Garlic
Coconut milk	Peanut butter	Canned fish
Galangal	Cheese	Cooked rice

Participants chose Munsell color chips to go with the odor stimuli. Eighty-four round color chips were mounted on a card, arranged by hue, value, and chroma in equal perceptual steps (cf. Majid, 2008; see Fig. S1 in the supplementary materials).

### Design and procedure

The participants completed two tasks. In the *odor–color task*, they had to smell an odor and choose a color from the color card. The color card was placed within reach of the participant to easily choose a color. The experimenter handled the bottles to prevent participants from guessing the contents (e.g., by shaking the bottle). The interval between two consecutive odors was the amount of time it took for the experimenter to exchange the bottle for a new one (approx. 25 s).

In the *odor-naming task*, participants were presented with the odors again and were asked to name each one and rate its familiarity on a 3-point scale (1 = *unfamiliar*, 2 = *somewhat familiar*, 3 = *familiar*). Familiarity was restricted to a 3-point scale to facilitate the task for the Maniq, who were not used to scales. The responses were audio-recorded for later transcription and coding.

Participants completed the odor–color task twice, separated by 2 h on average (range = 1–4 h), followed later by the odor-naming task. The order of the 15 odors was fixed within a task but varied across tasks.

## Results

Before examining the possible role language plays in odor–color associations, we first tested whether people consistently mapped the colors to odors. We looked at within-participant consistency, within-language consistency, and cross-language consistency. Thereafter, we examined the role of odor-naming strategies on odor–color mappings.

### Exclusion criteria

One Maniq participant completed the odor–color task only once and was therefore excluded. The naming data for one Dutch participant were not available, so that participant was not included in the later analyses involving naming data.

### Odor–color associations

#### Consistency within participants

**Fig. 1:**
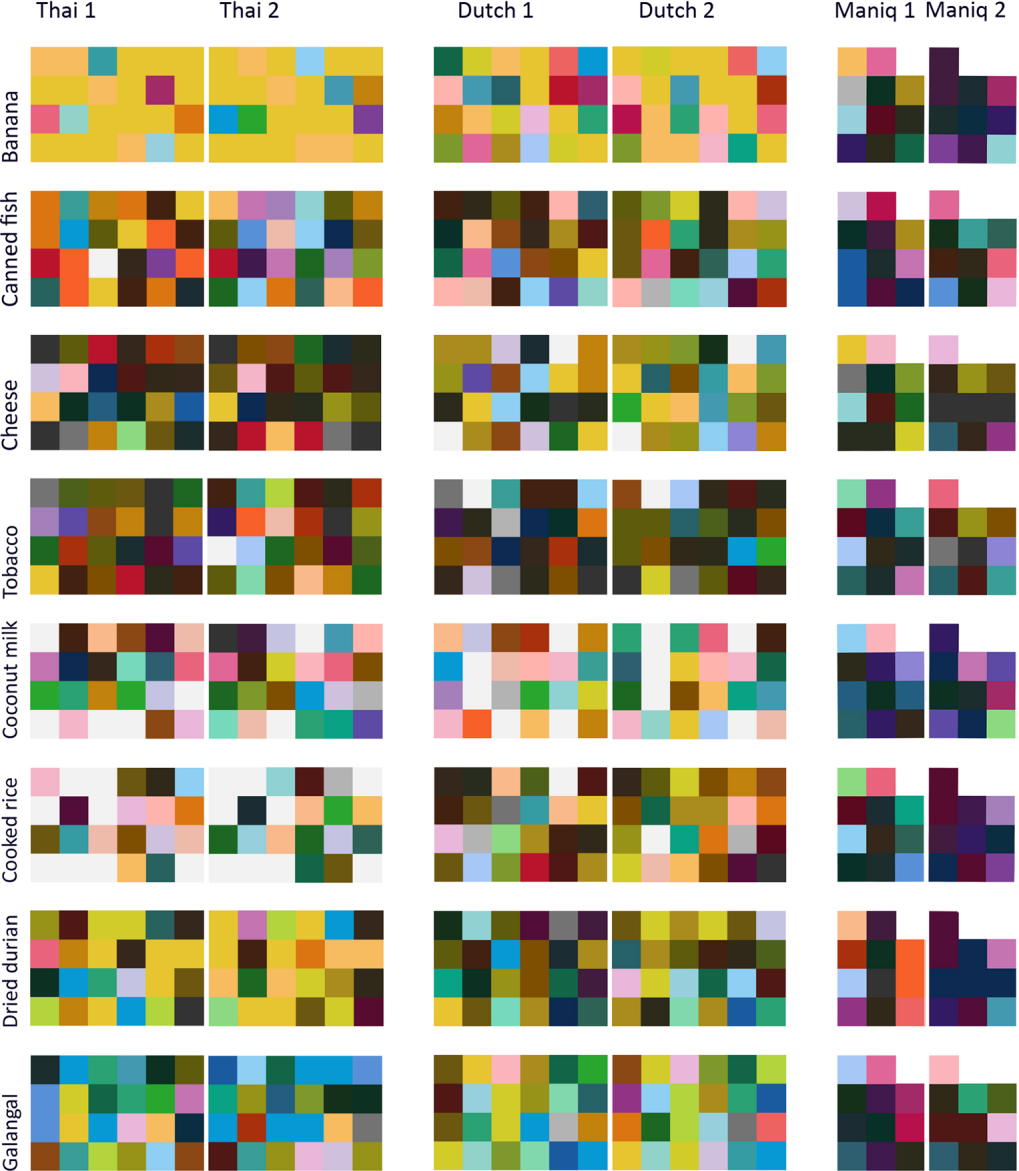

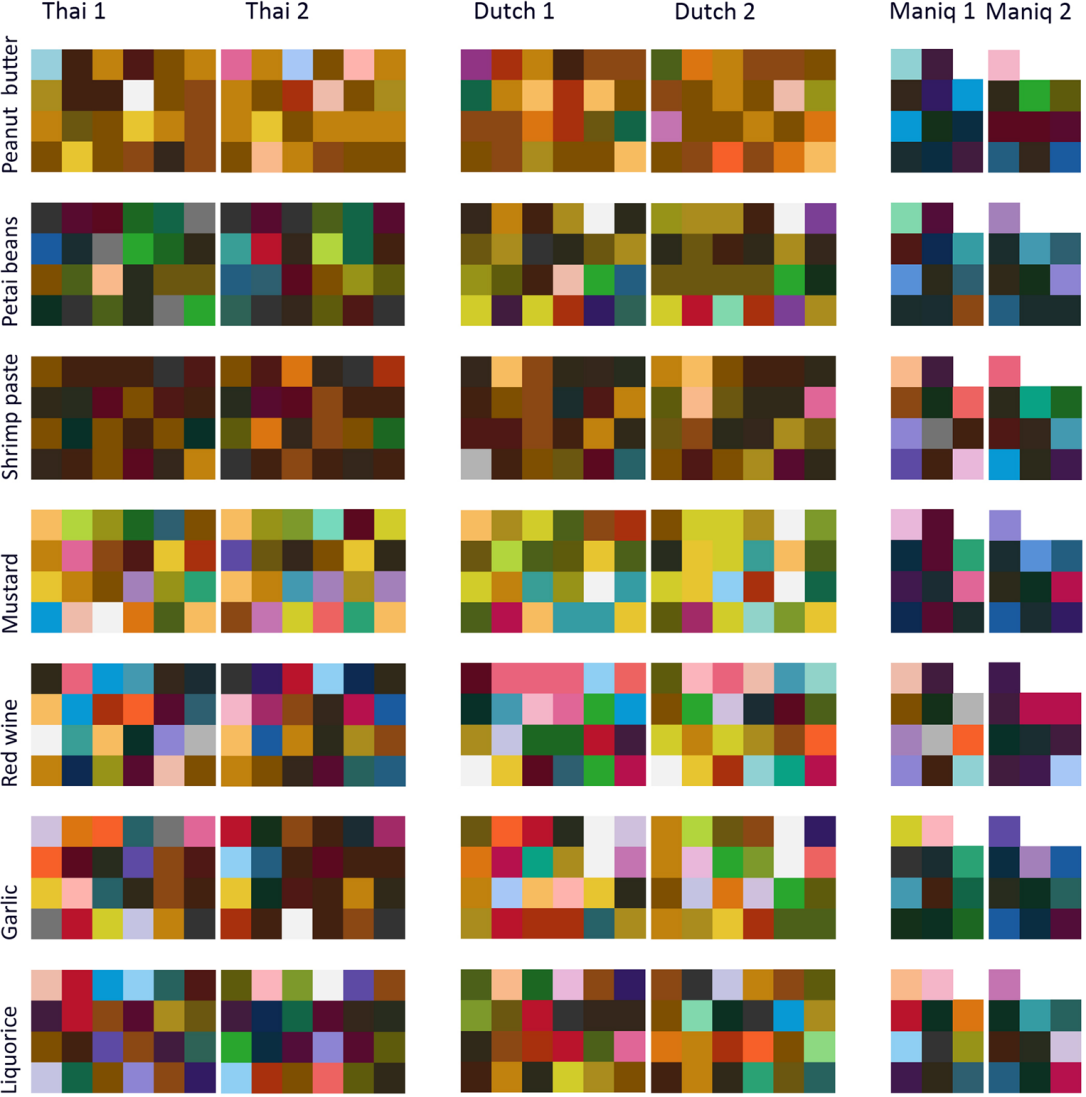
Plots of colors chosen for each odor by each individual speaker of Thai, Dutch, and Maniq at both times of testing (Time 1 and Time 2)

If participants randomly linked colors to odors across sessions, the expected chance of a consistent response (i.e., choosing the same color chip across the two sessions) would be $\frac{1}{\mathrm{number}\kern0.5em \mathrm{of}\kern0.5em \mathrm{color}\kern0.5em \mathrm{chips}}$. If, however, odor–color mappings are systematic, the proportion of observed consistent responses would be significantly higher. Binomial tests showed that both the Thai and Dutch were more consistent in their odor–color mappings than would be expected by chance (Thai: *N* = 360, observed proportion = .18, *p* < .001; Dutch: *N* = 360, observed proportion = .17, *p* < .001), but Maniq speakers were not (*N* = 150, observed proportion = .013, *p* = .27) (see Fig. 1; see also the supplementary materials).

#### Consistency within languages

If odor–color mappings were not consistent within a language, the expected chance within each session to choose a color chip would be $\frac{1}{\mathrm{number}\kern0.5em \mathrm{of}\kern0.5em \mathrm{color}\kern0.5em \mathrm{chips}}$. For each odor, as many binomial tests were performed as the number of colors chosen in that language and session; therefore, the Bonferroni-corrected alpha was $\frac{.05}{\mathrm{number}\kern0.5em \mathrm{of}\kern0.5em \mathrm{different}\kern0.5em \mathrm{color}\kern0.5em \mathrm{chips}\kern0.5em \mathrm{chosen}}$. Only odors chosen significantly more often than would be expected by chance are shown in Fig. 1. Robust associations (i.e., at least one color was selected more than would be expected by chance at both Times 1 and 2) are shown in Fig. 1A. In Maniq, none of the odor–color associations were stable. Some associations appeared to be significant, but only at one time point. Only banana and peanut butter were stable for both the Thai and Dutch participants; shrimp paste, rice, and dried durian were stable for the Thai speakers, whereas fermented petai beans and coconut milk were stable for the Dutch speakers.

#### Consistency across languages

**Fig. 2:**
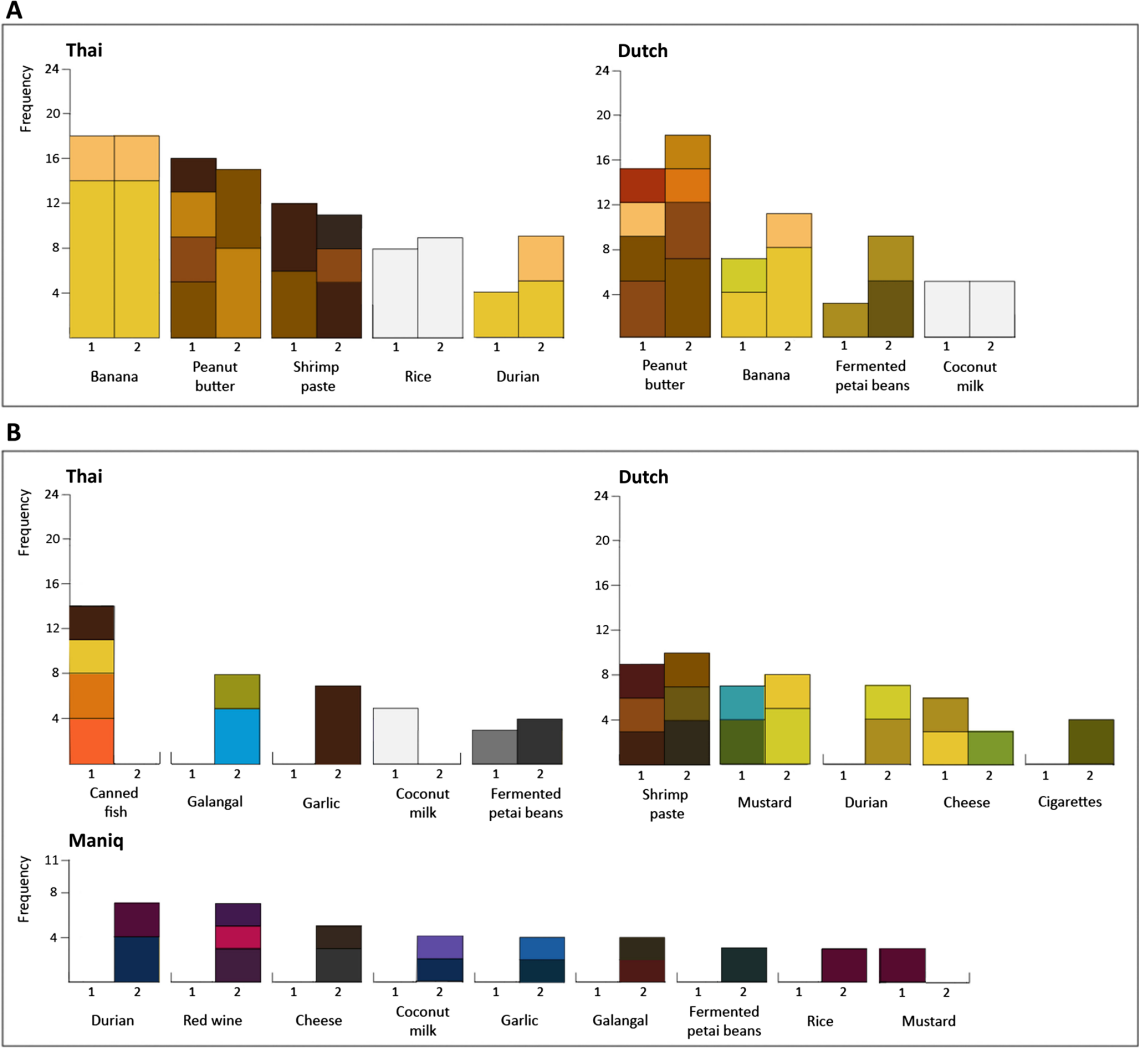
Odor–color associations chosen significantly above chance in Thai, Dutch, and Maniq. The frequency of participants who chose a particular color is shown along the *y*-axis, with the color at the bottom of a stack being the one most frequently selected. (**A**) Significant, stable odor–color associations (i.e., at least one color was significant at both Times 1 and 2). (**B**) Significant but unstable odor–color associations (i.e., odor–color associations significant at only one time point)

There were few consistencies across languages (see Figs. 1 and 2). For the seven odors with stable color associations over time, only two were stable in both Thai and Dutch: yellow tones were chosen for banana, and brown tones for peanut butter.

### Language and odor–color associations

#### Odor descriptions across languages

**Fig. 3:**
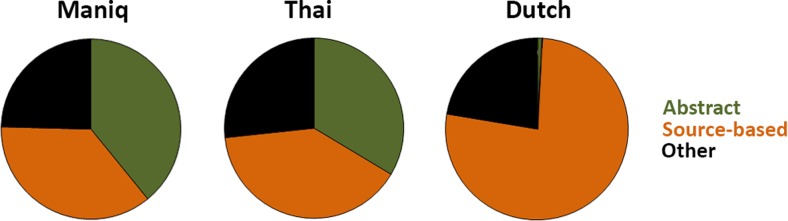
Dutch speakers relied predominantly on source-based terms to describe odors, but Maniq and Thai speakers used abstract terms as often as source-based terms

To investigate the possible role of language on odor–color associations, we first examined the odor descriptions in the odor-naming task, focusing on the main content descriptors. Modifiers and comments regarding the familiarity of the odor were excluded. The main responses were coded as “abstract” smell terms, “source-based” terms, or “other”. Abstract smell terms are not derived from an existing name of an odor source and are dedicated to smell quality (e.g., *stinky* in English; see Table 2). We coded as “source-based” terms all sources, regardless of the level of specificity (e.g., *banana*, *fruit*, *food*). The descriptors coded as “other” included references to taste (e.g., *sweet*), intensity (e.g., *strong*), touch (e.g., *soft*), and evaluations (e.g., *nice*). Following Majid and Burenhult (2014), first and all odor descriptions were analyzed separately. The preferred strategies to describe smells differed between groups. Dutch speakers overwhelmingly relied on source-based descriptions, but Maniq and Thai speakers used a mixture of abstract and source-based terms (see Fig. 3). This was true both in the first responses, *χ* ^2^(6) = 176.83, *p* < .001, Cramer’s *V* = 0.32, and in all responses, *χ* ^2^(6) = 239.68, *p* < .001, Cramer’s *V* = 0.32.

**Table 2:** Abstract smell descriptors used in the odor-naming task by Maniq, Thai, and Dutch speakers, with brief definitions and exemplars

Maniq Smell Terms	Definition (from Wnuk & Majid, 2014)	Example Stimuli in the Naming Task
*caŋɛs*	to smell like burnt animal fur, used also of roasted animal fat	cigarettes, coconut milk
*lspəs*	to smell fragrant, as of, e.g., medicinal plants, wild yams, bearcats, forest	dried durian, galangal, banana, coconut milk, cigarettes, licorice, peanut butter, wine, mustard, shrimp paste, cheese, garlic
*hamis*	to have a bad smell, used mainly with reference to the smell of the sun, believed to be perceptible in the atmosphere on particularly hot days	garlic, fermented petai beans, cigarettes
*haʔĩt*	to stink, as of, e.g., rotting carcass, certain animals (e.g., bats), some wild yams (e.g., *Dioscorea daunea*)	canned fish, wine, shrimp paste, fermented petai beans, cheese, mustard, peanut butter, coconut milk
*paʔɔ̃ʔ*	to smell bad, as of, e.g., rotting or damp bamboo, bamboo tubes for storing water, mud, urine, petai beans	shrimp paste
*kamɛh*	to have a strong smell, as of, e.g., various types of millipedes, dart poison, fruit bats	canned fish
*miʔ danɔw*	smell of, e.g., mushrooms, rotten wood, old shelters, animal bones	cooked rice
*miʔ huhũɸ*	smell of, e.g., snakes, soil, tuber-digging, sweat	canned fish, coconut milk, dried durian, shrimp paste, garlic
*miʔ bayɔ̃ɸ*	smell of, e.g., old shelters, soil, mushrooms, rotten leaves	fermented petai beans, galangal, banana
Thai Smell Terms	Dictionary Definition (Haas, 1964)	Example Stimuli in the Naming Task
*hǒom*	to be fragrant, odoriferous, sweet smelling	banana, galangal, dried durian, cooked rice, peanut butter
*měn*	to smell bad, stink, be foul-smelling	fermented petai beans, cheese, garlic, shrimp paste, licorice
*chǔn*	to be strong (of odors), pungent (as the odor of strong tobacco)	cigarettes, licorice, mustard, garlic
(*měn) àp*	to smell stuffy, have a stuffy odor (as a closed room)	cheese, dried durian
*měn khǐao*	to smell bad; according to some speakers, to have an odor of crushed green leaves (khǐao, “to be green”)	fermented petai beans
*měn prîao*	to smell unpleasantly sour (prîao, “to be sour”)	cheese
Dutch Smell Terms	Dictionary Definition (Geerts, 1993)	Example Stimuli in the Naming Task
*muf*	musty	cheese, dried durian, cooked rice
*weeïg*	sickly	cooked rice
*stinken*	to stink	fermented petai beans

#### Odor language and odor–color associations

To examine the relation between language and odor–color correspondences, we used mixed logit models (Jaeger, 2008) performed in R (R Development Core Team, 2013) using the lme4 package (Bates et al., 2014).

##### Does the type of odor description predict odor–color associations?

To test whether the odor description predicted the odor–color associations, a mixed logit model was conducted with the fixed factors Language (Maniq, Thai, Dutch), Type of Odor Description (source-based, abstract), and Familiarity Rating (unfamiliar, somewhat familiar, familiar), with participants and items included as random effects. “Other” descriptions were not considered in this model, since no specific prediction could be made for these responses. The dependent variables were (1) color consistency, in which a color choice was considered consistent if the same chip was chosen in both sessions, and inconsistent otherwise; and (2) color match, in which a color was considered a match if it reflected the actual color of the odor source in at least one of the sessions (e.g., banana → yellow). Three of the authors separately determined which color chips constituted possible matches. Disagreements were resolved by discussion.

The Thai (18.5 %) and Dutch (18.5 %) were more consistent in their odor–color associations than the Maniq (1.5 %), *β* = 0.87, *SE* = 0.28, *z* = 3.15, *p* = .002, confirming the results from the binomial tests. Consistency was also higher for more familiar stimuli, *β* = 0.54, *SE* = 0.19, *z* = 2.91, *p* = .004. The type of description did not predict consistency, however, *β* = 0.39, *SE* = 0.35, *z* = 1.10, *p* = .27 (the consistency for source-based responses was 15.8 %, vs. 14.4 % for abstract responses).

The pattern was different for color matches. Here the type of response significantly predicted the likelihood of a veridical color match. More color matches were found for odors described with a source-based term (35.5 %) than for those described with an abstract term (22.6 %), *β* = 1.06, *SE* = 0.30, *z* = 3.58, *p* < .001. We also observed a main effect of language, with Thai (36.3 %) and Dutch (37.7 %) participants selecting more matching colors than the Maniq (9.2 %), *β* = 0.81, *SE* = 0.20, *z* = 4.10, *p* < .001. Familiarity did not predict an odor–color match, *β* = 0.27, *SE* = 0.15, *z* = 1.78, *p* = .076.

##### Does the correctness of an odor description predict odor–color matches?

If odor–color associations are determined by a label, we might predict correctly identified sources would have more correct and consistent matches than incorrectly identified sources. Since “correctness” can only be determined for source-based descriptions, we ran a separate analysis on the source-based descriptions with the fixed factors Language (Maniq, Thai, Dutch), Correctness of Odor Description (correct, incorrect), and Familiarity Rating (unfamiliar, possibly familiar, familiar), controlling for random subject and item effects. The dependent variables again were (1) color consistency, that is, whether the same color chip was chosen in both sessions, and (2) color match, that is, whether the color choice reflected the actual color of the odor source. Participants were more consistent when their response was correct (27.0 %) than when it was incorrect (11.6 %), *β* = 0.65, *SE* = 0.31, *z* = 2.10, *p* = .036. Similarly, participants chose more matching colors to odors when their response was correct (63.1 %) than when it was incorrect (25.1 %), *β* = 1.36, *SE* = 0.28, *z* = 4.82, *p* < .001.

## Discussion

Describing odors is difficult (see, e.g., Cain, 1979; Engen, 1982; Olofsson & Gottfried, 2015; Yeshurun & Sobel, 2010), at least in Western languages (e.g., Majid & Burenhult, 2014). Therefore, until now the role of language in odor–color associations has not received much attention (e.g., Deroy et al., 2013; Levitan et al., 2014). The findings reported here, however, suggest language plays an important role in odor–color associations.

Unlike the Thai and Dutch participants, the hunter-gatherer Maniq showed few, if any, consistent or accurate odor–color correspondences. This could have been due to the smaller sample size for this group. However, an examination of the few reliable associations made by the Maniq suggests this is unlikely to be the whole story. The colors chosen by the Maniq were rarely even in the vicinity of the original odor sources (see Figs. 1 and 2), belying a simple explanation in terms of statistical power. It is possible, however, that the differences between the Maniq versus the Thai and Dutch are due to the adoption of a different strategy for performing the task, caused by environmental or cultural factors. Previous studies have shown urbanization, schooling, and other cultural practices can impact brain organization (Dehaene et al., 2010), influencing attention systems, amongst other things (Dehaene, Cohen, Morais, & Kolinsky, 2015; Linnell, Caparos, de Fockert, & Davidoff, 2013; Miyamoto, Nisbett, & Masuda, 2006). Future studies would have to examine each of these factors to disentangle the probable causes.

More pertinently, when people used abstract smell terms to describe the odors, they were less likely to choose a color match: The associations were more variable. When they described an odor with a source-based term, on the other hand, their color choices more accurately reflected the odor source. Moreover, the color associations were more veridical (i.e., reflecting the color of the source object in the real world), and more consistent, when the odor source was named correctly. This suggests an important strategy for assigning colors to odors is via language. A previous study (Lehrner, Glück, & Laska, 1999) had shown that people were more likely to describe an odor consistently when the odor name they had generated was correct. So, it is possible the correctly named odors in this study were also the ones whose labels were consistently retrieved in both odor–color tasks, and so the same color was chosen on both occasions.

Consistency over time often had not been taken into account in previous studies of odor–color associations (e.g., Demattè et al., 2006), but the data reported here indicate not all matches are equally robust (see also Gilbert et al., 1996; Stevenson et al., 2012). Of the odor–color associations that were stable over time, only two were reliable across groups. This confirms previous results suggesting considerable cross-cultural variation in cross-modal associations (e.g., Levitan et al., 2014). The substantial variation found between odors casts doubts on simple accounts of odor–color matches based solely on perceptual alignments (cf. Deroy et al., 2013; Spector & Maurer, 2012)

To conclude, many studies have reported links between odors and colors (e.g., Blackwell, 1995; Davis, 1981; Stevenson & Oaten, 2008; Zellner et al., 1991), but few have considered the role of language. We showed here that speakers of different languages, with different strategies for naming odors, differ in how they match colors to odors. Language, thus, matters for odor–color associations.

## Electronic supplementary material

Below is the link to the electronic supplementary material.

ESM 1 (DOCX 121 kb)
