# Supplementary material for: Odor–color associations differ with verbal descriptors for odors: A comparison of three linguistically diverse groups
Source: Psychon Bull Rev. 2016 Oct 25;24(4):1171–9. doi: 10.3758/s13423-016-1179-2 (PMC5570805; doi:10.3758/s13423-016-1179-2)
Supplement: Supplementary file 1 — (DOCX 121 kb) [file 13423_2016_1179_MOESM1_ESM.docx]

Supplementary Materials

Odor-Color Associations Differ with Verbal Descriptors for Odors: A Comparison of Three Linguistically Diverse Groups

Josje M. de Valk, Ewelina Wnuk, John L. A. Huisman, and Asifa Majid

Figure S: Color chart used in the odor-color association task (cf. Majid, 2008). Note the colors are only an approximation of the Munsell chart used in the actual experiment.

**
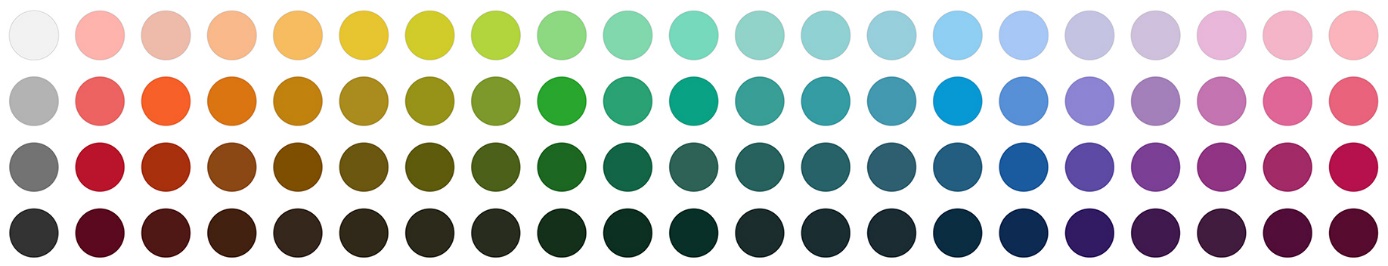
Consistency of Odor-Color Associations By Color Categories.**

In the main analyses, we tested whether participants were consistent in which colors they assigned to which odors over the two test sessions using a strict criterion of exact match. According to this test, Maniq speakers were not consistent in matching odors to colors. Here we ask whether a different result would be obtained if we used a more liberal criterion of consistency, where a consistent color response is one which is from the same color category.

It is known that color categories vary across cultures (Berlin & Kay, 1969; Malt & Majid, 2013; Regier & Kay, 2009), so we first established for each language their color terms for the colors used in this experiment using the methods from the Language of Perception survey (Majid, 2008; Majid & Levinson, 2007). In a separate test Thai, Dutch, and Maniq participants were asked to name the colors used in this experiment one-by-one. Accordingly, we were able to establish that Thai and Dutch both used 10 distinct color terms to exhaustively name the colors, whereas Maniq only used 6 terms (roughly *black, white, red, yellow, grue* and *purple*; see also Wnuk, 2016).

We tested if participants’ responses differed from chance where expected chance of a consistent response was $\frac{1}{number of color terms}$. Note, this further favors finding consistency in responses amongst the Maniq, and that is what we find *N* = 150, observed proportion = .273, *p <* .001. Binomial tests also confirmed both Thai and Dutch were more consistent in their odor-color mappings than expected by chance using color terms as criterion – Thai: *N* = 360, observed proportion = .397, *p* < .0001; Dutch: *N* = 360, observed proportion = .383, *p*< .0001.

References

Berlin, B., & Kay, P. (1969). *Basic color terms: Their universality and evolution*. Berkeley: University of California Press.

Majid, A. (2008). Focal colors. In *Field Manual* (Vol. 11, pp. 8–10). Nijmegen: Max Planck Institute for Psycholinguistics.

Majid, A., & Levinson, S. C. (2007). The language of vision I: Color. In *Field Manual Volume 10*. Nijmegen: Max Planck Institute for Psycholinguistics.

Malt, B. C., & Majid, A. (2013). How thought is mapped into words. *Wiley Interdisciplinary Reviews: Cognitive Science*, *4*(6), 583–597.

Regier, T., & Kay, P. (2009). Language, thought, and color: Whorf was half right. *Trends in Cognitive Sciences*, *13*(10), 439–446.

Wnuk, E. (2016). *Semantic specificity of perception verbs in Maniq* (PhD dissertation). Radboud University, Nijmegen.
